# Structural and Functional Characterization of New Lipid Transfer Proteins with Chitin-Binding Properties: Insights from Protein Structure Prediction, Molecular Docking, and Antifungal Activity

**DOI:** 10.1021/acs.biochem.4c00124

**Published:** 2024-07-05

**Authors:** Gabriella Rodrigues Gonçalves, Marciele Souza da Silva, Layrana Azevedo dos Santos, Thomas Zacarone Afonso Guimarães, Gabriel Bonan Taveira, Felipe Astolpho Almeida, Sarah Rodrigues Ferreira, Antonia Elenir Amancio Oliveira, Celso Shiniti Nagano, Renata Pinheiro Chaves, Vanildo Silveira, André de Oliveira Carvalho, Rosana Rodrigues, Valdirene Moreira Gomes

**Affiliations:** † Laboratório de Fisiologia e Bioquímica de Microrganismos, Centro de Biociências e Biotecnologia, Universidade Estadual do Norte Fluminense Darcy Ribeiro, 28013-602 Campos dos Goytacazes, RJ, Brazil; ‡ Laboratório de Química e Função de Proteínas e Peptídeos, Centro de Biociências e Biotecnologia, Universidade Estadual do Norte Fluminense Darcy Ribeiro, 28013-602 Campos dos Goytacazes, RJ, Brazil; § Laboratório de Bioquímica Marinha (BioMar-Lab), Departamento de Engenharia de Pesca, Universidade Federal do Ceará (UFC), 60455-900 Fortaleza, Ceará, Brazil; ∥ Laboratório de Biotecnologia, Centro de Biociências e Biotecnologia, Universidade Estadual do Norte Fluminense Darcy Ribeiro, Campos dos Goytacazes, 28013-602 RJ, Brazil; ⊥ Laboratório de Melhoramento e Genética Vegetal, Centro de Ciências e Tecnologias Agropecuárias, Universidade Estadual do Norte Fluminense Darcy Ribeiro, 28013-602 Campos dos Goytacazes, RJ, Brazil

## Abstract

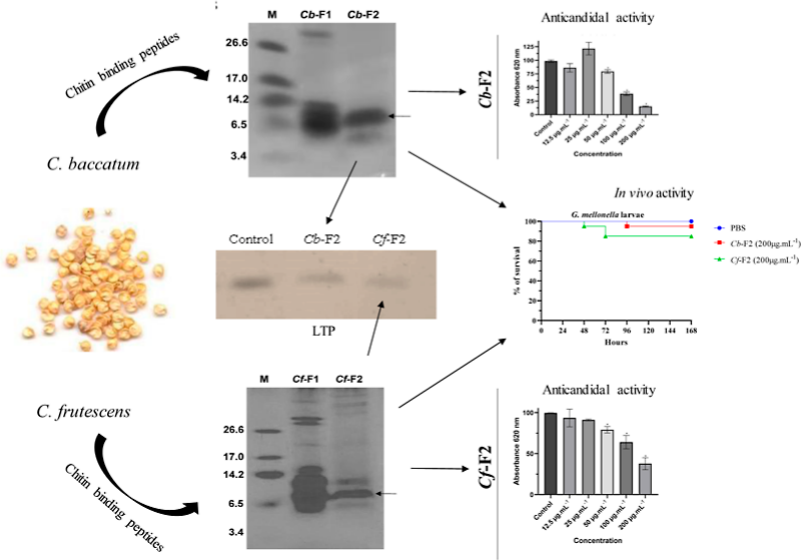

Faced with the emergence of multiresistant microorganisms that affect human health, microbial agents have become a serious global threat, affecting human health and plant crops. Antimicrobial peptides have attracted significant attention in research for the development of new microbial control agents. This work’s goal was the structural characterization and analysis of antifungal activity of chitin-binding peptides from *Capsicum baccatum* and *Capsicum frutescens* seeds on the growth of *Candida* and *Fusarium* species. Proteins were initially submitted to extraction in phosphate buffer pH 5.4 and subjected to chitin column chromatography. Posteriorly, two fractions were obtained for each species, *Cb*-F1 and *Cf*-F1 and *Cb*-F2 and *Cf*-F2, respectively. The *Cb*-F1 (*C. baccatum*) and *Cf*-F1 (*C. frutescens*) fractions did not bind to the chitin column. The electrophoresis results obtained after chromatography showed two major protein bands between 3.4 and 14.2 kDa for *Cb*-F2. For *Cf*-F2, three major bands were identified between 6.5 and 14.2 kDa. One band from each species was subjected to mass spectrometry, and both bands showed similarity to nonspecific lipid transfer protein. *Candida albicans* and *Candida tropicalis* had their growth inhibited by *Cb*-F2. *Cf*-F2 inhibited the development of *C. albicans* but did not inhibit the growth of *C. tropicalis*. Both fractions were unable to inhibit the growth of *Fusarium* species. The toxicity of the fractions was tested in vivo on *Galleria mellonella* larvae, and both showed a low toxicity rate at high concentrations. As a result, the fractions have enormous promise for the creation of novel antifungal compounds.

## 1 Introduction

All living organisms naturally produce antimicrobial peptides (AMPs), which are a class of molecules that act as protection against pathogens, including bacteria and fungus.^1^ They have a low molecular weight and net positive charge and are rich in hydrophobic amino acids.^2,3^ In plants, they are part of innate defense, serving as the primary defense against infections triggered by pathogenic microorganisms and are present in all plant organs.^4^ This group includes thionins, defensins, cyclotides, and lipid transport proteins (LTPs). Another class of plant-isolated proteins with antifungal properties is the chitin-binding proteins. These proteins bind to chitin by presenting chitin-binding domains (CBDs). This CBD, whose amino acid sequence is known, contains a common structural motif of 30–43 amino acids with several cysteines and glycines in conserved positions, also known as the hevein domain.^5^ The proteins that are part of this family do not bind solely and exclusively to chitin and can bind to several glycoconjugate complexes containing *N*-acetyl-d-glucosamine or *N*-acetyl-d-neuraminic acid.^5^ These proteins also have the ability to reversibly bind to different chitin matrices, such as the cell wall of fungi. Research has indicated that plant CBPs have antifungal properties against phytopathogens like *Fusarium oxysporum* and *Colletotrichum gloeosporioides*(6,7) and yeast of the genera *Candida*.^8^ This group includes proteins like hevein, chitinases, lectins, and more recently, 2S albumin. In 2024, the presence of a 2S albumin from *Capsicum annuum* seeds with chitin-binding properties was reported for the first time. This protein showed antimicrobial activity against *Candida albicans* and *Candida tropicalis* and was named *Ca*-Alb2S.^9^ To date, the presence of chitin-binding LTPs has not been reported.

LTP, also known as a nonspecific lipid transfer protein (nsLTP), is produced by several types of plants. LTPs are quite abundant, rich in cysteine residues with conserved positions, are secretory and soluble, and have a molecular mass of less than 10 kDa.^10−13^ Structurally, LTPs have four to five α-helices, which are stabilized through four relatively conserved disulfide bonds with the pattern of C–Xn–C–Xn–CC–Xn–CXC–Xn–C–Xn–C. The folding of the helix creates a central hydrophobic groove ideal for interacting with a variety of lipids, such as fatty acids and phospholipids.^14^ Based on their molecular weight, LTPs are divided into two groups: LTP type 1, which has approximately 7 kDa, and LTP type 2, which has approximately 10 kDa.^15^ The disulfide bonding pattern in type 1 is C1–C6 and C5–C8, whereas in type 2, C1–C5 and C6–C8 possibly differ in the tertiary structure; in type 1, there is a lengthy tunnel-like hydrophobic cavity, whereas in type 2, LTPs consist of two hydrophobic cavities positioned head-to-head.^16,17^

LTPs perform several functions simultaneously. One of the most reported functions is the defense function in plants against pathogens. It is feasible that the reaction mechanism includes the apoplast’s nsLTP secretion, so that they bind to lipid molecules secreted by the plants or to the molecules secreted by the microorganism.^18^ nsLTPs are also involved in biological processes such as fruit ripening,^19^ seed growth and germination,^18,20^ lipid barrier assembly,^14,18^ and lipid transport^21^ and are also associated with abiotic stress in plants, demonstrating that its overexpression increased tolerance in a saline stress environment.^22^ Furthermore, there are no reports of nsLTP exhibiting toxicity against plant or animal cells.

Several works have reported the isolation of this biomolecule in different species of plants and in different organs. Schmitt et al.^23^ isolated BrLTP2.1, which was extracted from the nectar of *Brassica rapa*, and its antifungal activity was reported. McLTP1 was extracted from *Morinda citrifolia* seeds showing antibacterial and anti-inflammatory activities and improved survival in lethal sepsis.^24,25^ The ajwain nsLTP1 protein isolated from *Trachyspermum ammi* seeds has also been described, but there is no description of antimicrobial activities.^26^

Regarding plants of the *Capsicum* genus, we can highlight that they were identified in several plant organs of this genus, including fruits, seeds, and leaves. We can mention *Ca*-LTP1 isolated from *Capsicum annuum* seeds, which showed inhibitory properties and activity against human salivary α-amylase. We can also mention *Cc*LTP isolated from *Capsicum chinense* fruits, but its biological activity has not been described.^27^

Antimicrobial resistance continues to grow in the population, increasing the demand for new bioactive molecules that have a wide spectrum of activities. Thus, it is crucial to find new antifungal agents, especially ones that are produced by the plants themselves.^28^ In this scenario, research on plant chitin-binding proteins with applications in the medical field was intensified. Therefore, this work’s goal was to identify and characterize chitin-binding peptides from the seeds of *Capsicum baccatum* (accession UENF 1732) and *Capsicum frutescens* (accession UENF 1775) and to analyze their possible biological activities.

## 2 Materials and Methods

### 2.1 Plant Material

The seeds of *C. baccatum* (access UENF 1732) and *C. frutescens* (access UENF 1775) were provided by the Laboratory for Plant Genetic Improvement, Center for Agricultural Sciences and Technologies, UENF, Brazil. The seeds were sown in 72-cell polystyrene trays filled with a substrate fertilized with a 4N/14P/8K formulation (Vivatto). These trays were kept in a growth chamber at 28 °C and watered once daily. Following the appearance of two sets of true leaves, each seedling was moved into a 5 L plastic pot with a 2:1 soil-to-substrate ratio. The pots were then transferred to a climate-controlled greenhouse. The plants continued to receive daily irrigation until the fruits matured and were subsequently harvested.

### 2.2 Microorganisms

The yeast species *C. albicans* (CE022) and *C. tropicalis* (CE017) and the filamentous fungi *F. oxysporum* and *F. solani* were grown in Sabouraud medium and kept at 4 °C in the Laboratory of Physiology and Biochemistry of Microorganisms of the Center for Biosciences and Biotechnology of the UENF, Brazil.

### 2.3 Protein Extraction and Precipitation with Ammonium Sulfate

Initially, 5 g of seeds was pounded into an extremely fine-grained flour. As soon as the flour was acquired, the proteins were submitted to extraction. Proteins were extracted from the flour using the procedure outlined by Ribeiro et al.^9,29^

### 2.4 Protein Quantification

Protein calculations were carried out quantitatively using the bicinchoninic acid method,^30^ with ovalbumin (Sigma-Aldrich) used as the standard protein.

### 2.5 Isolation of Proteins and Peptides with Affinity for Chitin

Poly(1–4)-β-*N*-acetyl-d-glucosamine, also known as chitin, was extracted from shrimp shells and is available commercially from Sigma-Aldrich in powder form. First, chitin underwent a chemical treatment as described by Miranda et al.^31^ Initially, 500 mL of 100 mM HCl was used to acidify 25 g of industrial chitin, which was then incubated for 24 h at 4 °C with intermittent shaking. Following the incubation period, the precipitate was treated with 250 mL of 100 mM NaOH after HCl was completely removed. This mixture was then heated at 100 °C for 16 h. Following heating, NaOH was removed, and an additional 250 mL of NaOH was added for another 16 h heating period. This step was repeated once more, resulting in three 16 h heating cycles in total. After the final heating, all NaOH was removed, and 200 mM HCl was added to the precipitate. Following the last heating, the precipitate was mixed with 200 mM HCl after all of NaOH had been removed. Following this last acidification, the precipitate was mixed with distilled water for storage after HCl was removed. The chitin column was then filled with 50 mg of the heated, peptide-rich fraction that had been solubilized in the same buffer after it had been equilibrated with 0.1 M sodium acetate buffer (pH 5.5). Equilibration buffer was initially used for chromatography. After that, the retained fraction was eluted using a 0.05 M HCl solution, and the desorbed proteins’ absorbance was measured at 280 nm. A flow rate of 1 mL/min was employed. Dialysis and lyophilization were followed by the collection and recovery of protein peaks.

### 2.6 Tricine Gel Electrophoresis in the Presence of SDS

Tricine–SDS–PAGE was performed using glass plates of 8 × 10 and 7 × 10 cm, together with 0.75 mm spacers. The separating gel was prepared with 16.4% acrylamide/bis-acrylamide concentration, and the stacking gel concentration was 3.9%. After 5 min of heating at 100 °C, the samples underwent a 5 min centrifugation at 15,000*g*. Thereafter, each sample was added to the gel at a concentration of 20 μg.mL^–1^. For around 16 h, electrophoresis was operated at a steady voltage of 24 V. M3546 (Sigma-Aldrich) was used as a protein molecular weight marker, with molecular masses of 26.600, 17.000, 14.200, 6.500, 3.496, and 1.060 kDa.^32^

### 2.7 Western Blotting

Western blotting was carried out using the procedures outlined by Towbin et al.^33^ Following electrotransfer, the membranes were incubated for 16 h at 4 °C in a blocking buffer containing 2% skim milk powder. After that, the membranes were rinsed 10 times at room temperature for 5 min each time in phosphate-buffered saline (PBS) (10 μM NaH_2_PO_4_, 0.15 M NaCl, pH 7.4). Subsequently, a primary anti-LTP antibody (1:1000) was cultivated on the membranes in the blocking buffer. After another round of washing, the membranes were incubated with a secondary antibody (1:500) in blocking buffer for 1 h at room temperature with gentle agitation and washed again as previously described. 3,3′-Diaminobenzidine (DAB) was used to develop the membranes until the stained bands were visible. The developing solution included 40 μM tris–HCl, pH 7.5, 1 mg/mL^–1^ DAB, 100 μM imidazole, and 0.03% hydrogen peroxide.

### 2.8 In-Gel Digestion

Using a scalpel, the gel bands were cut into slices and then into tiny cubes of ∼1 mm^3^. Each cubed slice was placed in a separate 1.5 mL tube, and 1000 μL of destaining solution (50 mM AmBic/50% ACN—1:1) was added to each sample. The tubes were gently agitated on a Thermomixer at room temperature overnight. After removing the solution, 200 μL of fresh destaining solution was added for 1 h before removal. The gel bands were then dehydrated by adding 500 μL of 100% ACN to each tube for 1 min, followed by a repeated step. For protein reduction, 200 μL of a solution containing 10 mM DTT/100 mM AmBic was added to each tube and incubated at 55 °C for 30 min with gentle agitation on a Thermomixer. Subsequently, 500 μL of 100% ACN was added to each tube for dehydration, followed by the addition of 200 μL of alkylation solution (55 mM iodoacetamide––IAA/100 mM AmBic). The tubes were kept in the dark on a Thermomixer at room temperature for 30 min.

For protein digestion, 200 μL of cold trypsin solution (digestion solution containing trypsin in 10 mM AmBic/10% ACN) was used. The tubes were kept at 4 °C for 30 min and then transferred to a Thermomixer at 37 °C overnight for complete digestion. Then, 200 μL of extraction buffer (containing 1:2 of 5% formic acid to 100% ACN) was added, and the mixture was incubated at 37 °C for 30 min in a Thermomixer. The samples were then evaporated in a SpeedVac until completely dry. Prior to mass spectrometry analysis, they were resuspended in 50 μL of 0.1% formic acid in 50 mM AmBic.^34^

### 2.9 Mass Spectrometry Analysis

The Marine Biochemistry Laboratory (BioMar-Lab), Department of Fisheries Engineering, Federal University of Ceará (UFC), Ceará, Brazil, collaborated with us to identify the peptides found in *Cb*-F2. Tricine–SDS–PAGE was used to recover one band from *Cb*-F2, which was followed by the extraction of tryptic peptides^33^ and LC–MS/MS analysis. A hybrid mass spectrometer (ESI-Q-ToF) (Synapt HDMS, Waters Corp, MA, USA) was the instrument utilized. The data collection and processing parameters were analyzed using matrix-assisted laser desorption (ESI-Quad-ToF) mass spectrometry.^35^ The Mascot tool was used to evaluate the spectra, and the sequenced peptides were aligned using the BLASTp tool.^36^ Sequences with a high percentage of identity were selected and subjected to multiple alignments using CLUSTAL Multiple Sequence Alignment via the MUSCLE program (version 3.8).^37^ Merely, the residues detected using mass spectrometry were taken into account for calculating the percentages of identical and positive residues.

To identify the peptides present in *Cf*-F2, ESI–LC–MS/MS analyses were conducted at the Unit of Integrative Biology, Genomic and Proteomics Sector (UENF). A nanoAcquity UPLC system coupled to a Synapt G2-Si HDMS mass spectrometer (Waters, Manchester, United Kingdom) was utilized for the analysis. The samples were put through a nanoAcquity HSS T31 1.8 μm (75 μm × 150 mm) reversed-phase analytical column at 400 nL/min and 45 °C after first being placed onto a nanoAcquity UPLC 5 μm C18 trap column (180 μm × 20 mm) at a flow rate of 5 μL/min for 3 min. A binary gradient comprising mobile phase A (water and 0.1% formic acid) and mobile phase B (acetonitrile and 0.1% formic acid) was used to elute peptides. The gradient elution profile was initiated at 7% B, ramped to 40% B by 92.72 min, maintained at 99.9% B until 106.00 min, decreased to 7% B by 106.1 min, and remained at 7% B until the end of the 120 min experiment. Positive resolution mode (V mode) at 35,000 fwhm with ion mobility and independent data acquisition mode (HDMSE) were used for mass spectrometry. The ion mobility wave was set to a velocity of 600 m s^–1^, and the transfer collision energy ranged from 19 to 55 V in high-energy mode. A temperature of 70 °C was maintained at the source, while the cone and capillary voltages were kept at 30 and 2750 V, respectively. For the TOF parameters, the scan time was 0.5 s in continuum mode, covering a mass range from 50 to 2000 Da. Human [Glu1]-fibrinopeptide B (Sigma-Aldrich) at a concentration of 100 fmol/μL^–1^ was utilized as an external calibrant, with lock mass acquisition performed every 30 s. Mass spectra were acquired using MassLynx v4.0 software.^38^

### 2.10 Proteomic Data Analysis

The ProteinLynx Global Server (PLGS; version 3.0.2) (Waters, USA) and ISSO Quant workflow software were used for spectral processing and database searching.^39,40^ Three distinct thresholds were used for the PLGS analysis: 150 (counts) for low energy, 50 (counts) for increased energy, and 750 (counts) for intensity. Additionally, the following parameters were used in the analysis: allowance for two missed cleavages, a minimum of three fragment ions per peptide, a minimum of seven fragment ions per protein, and a minimum of two peptides per protein. Oxidation and phosphoryl were regarded as changeable modifications, whereas carbamidomethyl was designated as a fixed modification. The maximum rate of false discovery was set at 1%. Proteomic data were processed against the *C. annuum* protein database from UniProt (https://www.uniprot.org/proteomes/UP000222542) for *C. frutescens*.

### 2.11 Protein Structure Analysis

The predicted structural model for the identified proteins was generated using the FASTA sequences in the AlphaFold Protein Structure Database. AlphaFold is an AI system that predicts a protein’s 3D structure from its amino acid sequence. The generated models were further edited using the UCSF Chimera molecular graphics program (https://www.rbvi.ucsf.edu/chimera) to highlight the regions of interest in the protein structure.

### 2.12 Docking of LTP with the Tetramer of *N*-Acetylglucosamine (NAG)^4^

The DockThor v.2 program was used to dock LTP with (NAG)_4_.^41^ A blind docking approach was employed, defining a search space with a 40 × 40 × 40 Å cube covering 100% of the protein surface. Using PyMol and the Azahar plug-in for the oligosaccharide design, the (NAG)_4_ model was created. During docking, all rotations of the ligand were allowed while maintaining the rigidity of the protein atoms. For each experiment, the site’s usual search algorithm was used with 1,000,000 evaluations and 24 executions. Affinity energy values were used to determine the ideal LTP–ligand complex, which was then further examined utilizing the PLIP Web Tool program^42^ and the LigPlot + v program. 2.2.4.^43^ These algorithms detected noncovalent interactions between lipid transfer proteins (LTPs) and NAG.^4^

### 2.13 Effect of Peptides on Fungal Growth

The preparation of the inocula was carried out following the methodology previously described by Gonçalves et al.^9^ Initially, samples containing colonies of *C. albicans* and *C. tropicalis* were taken out of Petri dishes using a seeding loop. After that, these samples were put on brand new Petri dishes with Sabouraud agar (10 g/L peptone, 40 g/L d(+)glucose, 15 g/L agar) (Merck). The freshly made plates were baked for a full day at 30 °C. The cells were removed from the Petri dishes after incubation and homogenized in 10 mL of Sabouraud broth (10 g/L peptone, 20 g/L d(+) glucose) (Merck). A Neubauer chamber was used for cell quantification (LaborOptik) with an optical microscope (Axiovison A2, Zeiss). Inocula for filamentous fungi were taken from stocks and cultivated for 11 days at 30 °C in Petri dishes using Sabouraud agar (Merck). The spores were collected and homogenized in 10 mL of Sabouraud broth following growth in order to be quantified in a Neubauer chamber. Yeast (1 × 10^4^ cells/mL^–1^) and fungal cells (1 × 10^3^ cells/mL^–1^) were then incubated in Sabouraud broth supplemented with various concentrations of seed-derived fractions. The assays were conducted in 96-well cell culture microplates at 30 °C, with yeast evaluated over 24 h and filamentous fungi evaluated over 36 h. Cell growth was monitored by optical density readings taken every 6 h at a wavelength of 620 nm using a microplate reader. Each experiment was conducted in triplicate. The entire procedure was carried out in a laminar flow hood under aseptic conditions, following a methodology adapted from Broekaert et al.^44^ Cell growth in the absence of added proteins served as a control for comparison.

### 2.14 Cell Viability Analysis

The assay was carried out following the methodology previously described by Gonçalves et al.^9^ In order to assess the impact of *Cb*-F2 and *Cf*-F2 on the viability of yeast cells of *C. albicans* and *C. tropicalis*, a growth inhibition experiment was first conducted in order to generate colonies. Following a 24 h incubation period, the test cells (containing *Cb*-F2 and *Cf*-F2) and the control cells (lacking *Cb*-F2 and *Cf*-F2) were diluted for plating 1000×. Using a Drigalski loop, a 60 μL aliquot of the diluted solution was equally distributed across the surface of a Petri dish holding Sabouraud agar. After that, the dish was incubated for 36 h at 30 °C. Following this incubation time, the number of colony-forming units was counted, and the Petri plates were photographed. These experiments were conducted three times, and the results are presented with the assumption that the control group represents 100% cell viability.^45^

### 2.15 Effect of *Cb*-F2 and *Cf*-F2 Toxicity on *Galleria mellonella* Larvae

Larvae that weighed between 250 and 300 mg were chosen for this experiment, and 20 last instar *G. mellonella* larvae were utilized for the control and treatments. To inject 10 μL of *Cb*-F2 and *Cf*-F2 (200 μg/mL^–1^) into the larval hemocoel through the last probe, a Hamilton syringe was used. Two control groups were used: one group was inoculated with PBS, and the other group suffered only injury caused by the injection needle. The larvae were then put in Petri dishes and left to incubate for 7 days at 37 °C. Every 24 h, the number of dead larvae was recorded, and the larvae were considered deceased if they displayed no movement upon contact. Survival curves as percentages were generated, and differences in survival estimates were analyzed using the Kaplan–Meier method, along with log rank Mantel–Cox and Breslow–Wilcoxon tests. Software from GraphPad Software, Inc., California, CA, USA, was used for these analyses. The assay was conducted in duplicate.^46^

### 2.16 Statistical Analysis

One-way ANOVA was used to analyze the results of experiments measuring the suppression of filamentous fungal and yeast growth. Mean differences were deemed significant when the *p*-value was less than 0.05. GraphPad Prism software (version 5.0 for Windows) was utilized for all the statistical analyses.

## 3 Results

### 3.1 Chitin Affinity Chromatography, Electrophoretic Profile, and Detection of LTP by Western Blotting

**Figure 1:**
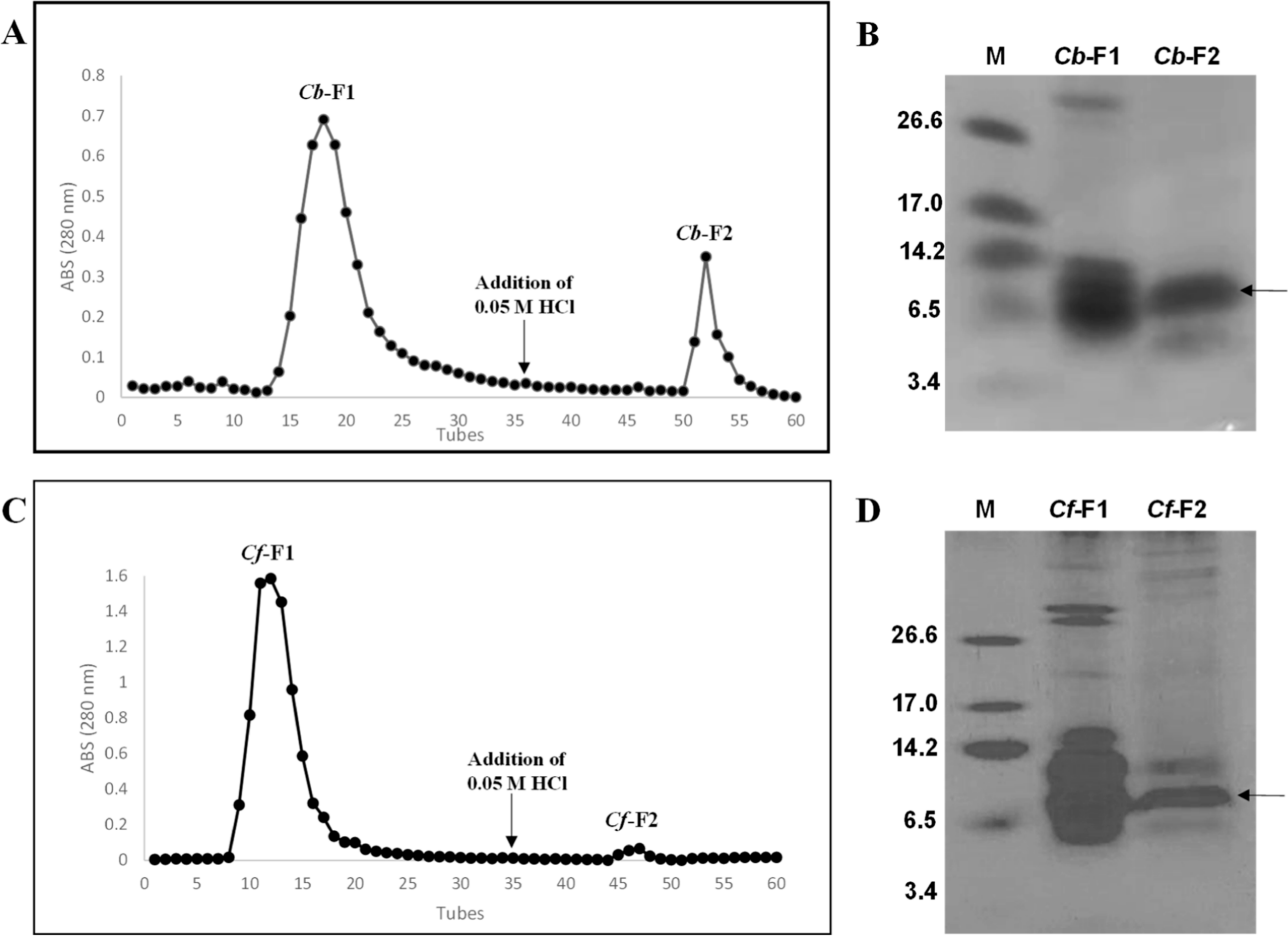
Chromatograms derived from chitin affinity chromatography of *C. baccatum* (A) and *C. frutescens* (C). Protein elution was monitored by absorbance at 280 nm. The flow was 0.5 mL/min. Electrophoretic visualization by tricine–SDS– PAGE of peptide-enriched fractions. *Cb*-F1 (1) and *Cb*-F2 (2): *C. baccatum* (B) and *Cf*-F1 (1) and *Cf*-F2 (2): *C. frutescens* (D). M: low molecular mass marker (kDa). The bands that underwent mass spectrometry are shown by arrows.

The chromatogram reveals that protein extracts from the *C. baccatum* and *C. frutescens* species were fractionated, yielding two major peaks for each species (Figure 1). The fractions called *Cb*-F1 (*C. baccatum*) and *Cf*-F1 (*C. frutescens*) did not bind to the chitin column. The fractions named *Cb*-F2 (*C. baccatum*) and *Cf*-F2 (*C. frutescens*) were the fractions that bound to the chitin column (Figure 1A,C). Thus, the presence of peptides that have the ability to bind to chitin is verified.

The fractions recovered from chromatography can be seen to have an electrophoretic profile in Figure 1B,D. In Figure 1B, which indicates the electrophoretic profile of *C. baccatum*, we can observe the protein bands for both fractions. *Cb*-F1 has bands in the marker range between 3.4 and 26.6 kDa, but there is a band above the 26.6 kDa marker. For *Cb*-F2, two major protein bands are demonstrated, between 3.4 and 14.2 kDa (Figure 1B). For *C. frutescens*, we can observe the electrophoretic profile in Figure 1D. In *Cf*-F1, we can observe major bands in the molecular marker range and bands above the 26.6 marker. For *Cf*-F2, we observed major protein bands between 6.5 and 14.2 kDa (Figure 1D).

**Figure 2:**
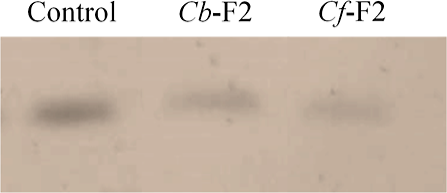
Western blotting of F2 fraction proteins extracted from different *Capsicum* species, *C. baccatum* (*Cb*-F2) and *C. frutescens* (*Cf*-F2), revealed by anti-LTP antibody. The positive control consisted of an LTP-rich fraction of *C. chinense* seeds.

The presence of LTP in the *Cb*-F2 and *Cf*-F2 fractions was demonstrated using anti-nsLTP serum by Western blotting. The control used was an LTP of *C. chinense* seeds (Figure 2).

### 3.2 Peptide Identification by Mass Spectrometry

**Figure 3:**
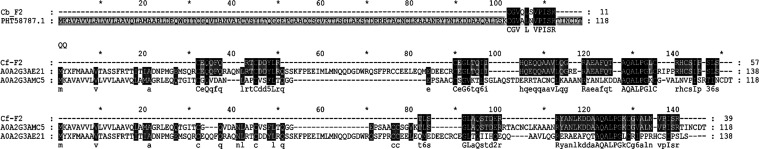
Alignment of amino acid residues of the peptides of the major protein bands from *Cb*-F2 and *Cf*-F2 seeds obtained by chitin affinity chromatography (protein band marked with an arrow in Figure 1B,D) showed similarity with the LTP protein type 1. Gray highlights represent regions of similarity, while black highlights represent identical regions.

For the identification of peptides obtained in the *Cb*-F2 and *Cf*-F2 fractions, major bands obtained by electrophoresis (20 μg) were submitted to mass spectrometry. The bands subjected to the technique are identified by an arrow in Figure 1B,D. The spectra were interpreted by Mascot and PLGS software, and fragments of peptide residue sequences were obtained. The residues were searched through the NCBI and UniProt BLASTP databases for proteins that were comparable to them. The obtained peptides CGVQLSVPISR (*C. baccatum*), TLSGLAQSTDERRYANLKDDAAQALPGKCGVALNVPISR, and CEQQFQRTCDDYLRCEGLTQIIHQEQQAAVLQGRAEAFQTAQALPGLCRHCSIPSLS (*C. frutescens*) were similar to the type 1 lipid transfer protein (LTP-1) (Figure 3), and the results were confirmed by Western blotting.

### 3.3 Three-Dimensional Structure

**Figure 4:**
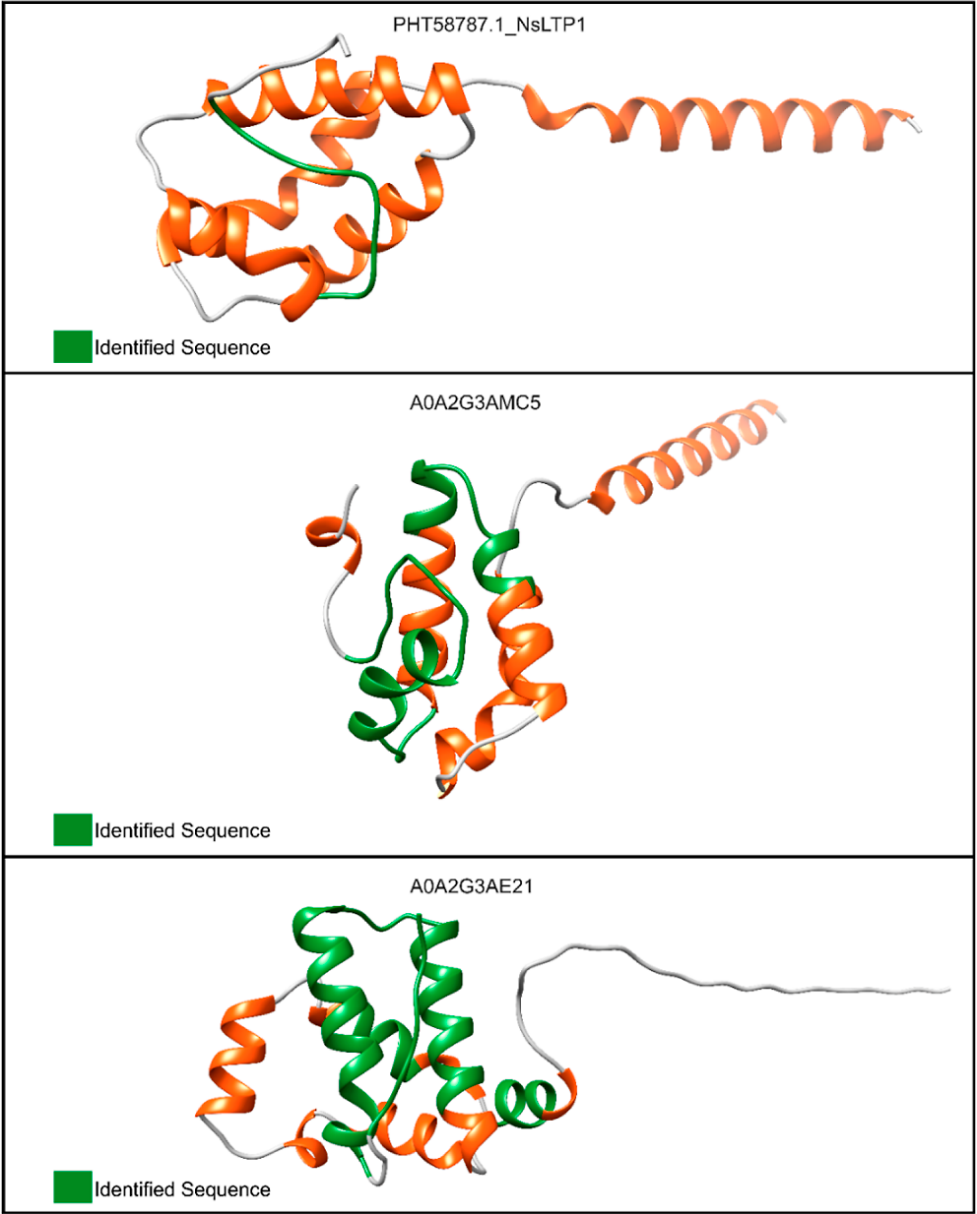
Three-dimensional structure of the proteins identified in this work. PHT58787.1_NsLTP1, A0A2G3AE21, and A0A2G3AMC5 refer to nonspecific nsLTPs. The FASTA sequences of each identified protein were used as input for protein modeling in the SWISS-MODEL server. The PHT58787.1_NsLTP1 accession was obtained from NCBI for *C. baccatum*. Both A0A2G3AE21 and A0A2G3AMC5 were obtained from the UniProt database for *C. annuum*. The peptide sequences identified in this work are highlighted in green. The structures colored in orange refer to α-helix regions, while the gray color refers to the coil regions.

Three LTPs were identified in this work, one from *C. baccatum* and two from *C. frutescens*. Based on the amino acid sequence, a search was performed on NCBI for *C. baccatum* and the UniProt database for *C. annuum*, where we performed a prediction of the three-dimensional structure of the peptides (Figure 4). For all proteins, in green, we have the region identified from mass spectrometry; in orange, we have an area highlighted that concerns the alpha-helices found, while the regions in gray represent the coil regions.

### 3.4 Docking of the LTPs with (NAG)_4_

**Figure 5:**
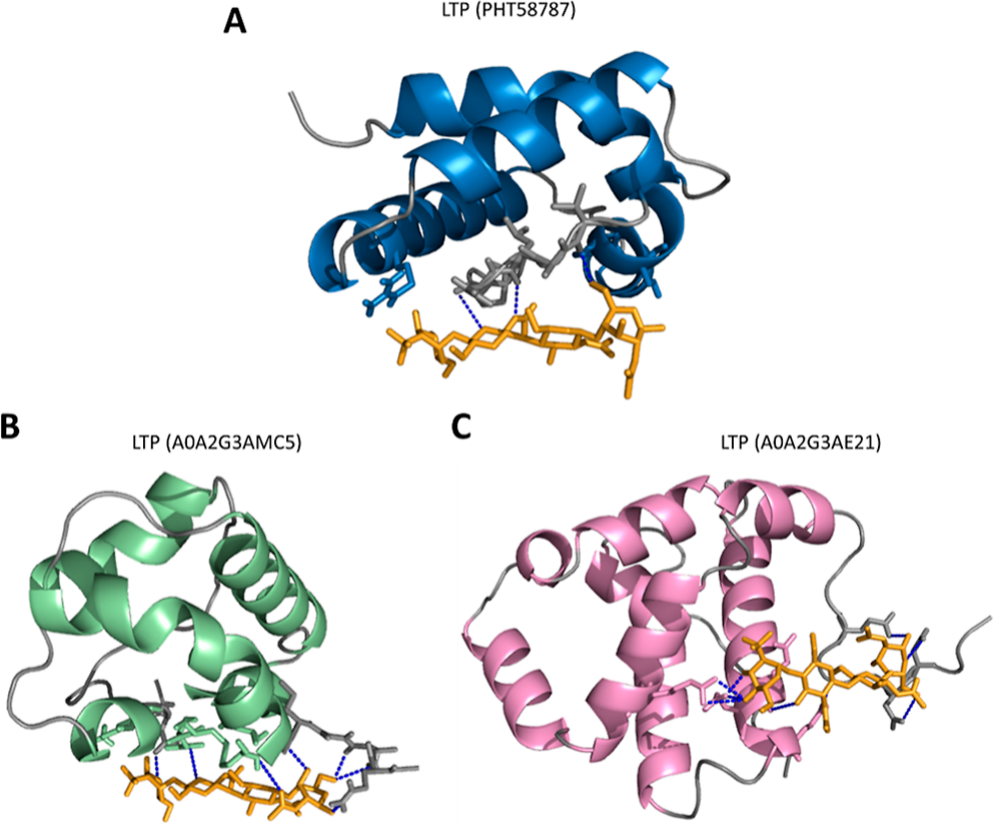
Three-dimensional structure of the LTP protein interacting with (NAG)_4_. A: LTP with similarity to *Cb*-F2, *C. baccatum*, accession: PHT58787. Alpha helices are represented in blue; loop regions are represented in gray. B: LTP with similarity to *Cf*-F2, *C. frutescens*, accession: A0A2G3AMC5. Alpha helices are represented in green; loop regions are represented in gray. C: LTP with similarity to *Cf*-F2, *C. frutescens*, accession: A0A2G3AE21. Alpha helices are represented in pink; loop regions are represented in gray. The NAG tetramer is represented in orange. Dashed blue lines = hydrogen bonds.

**Figure 6:**
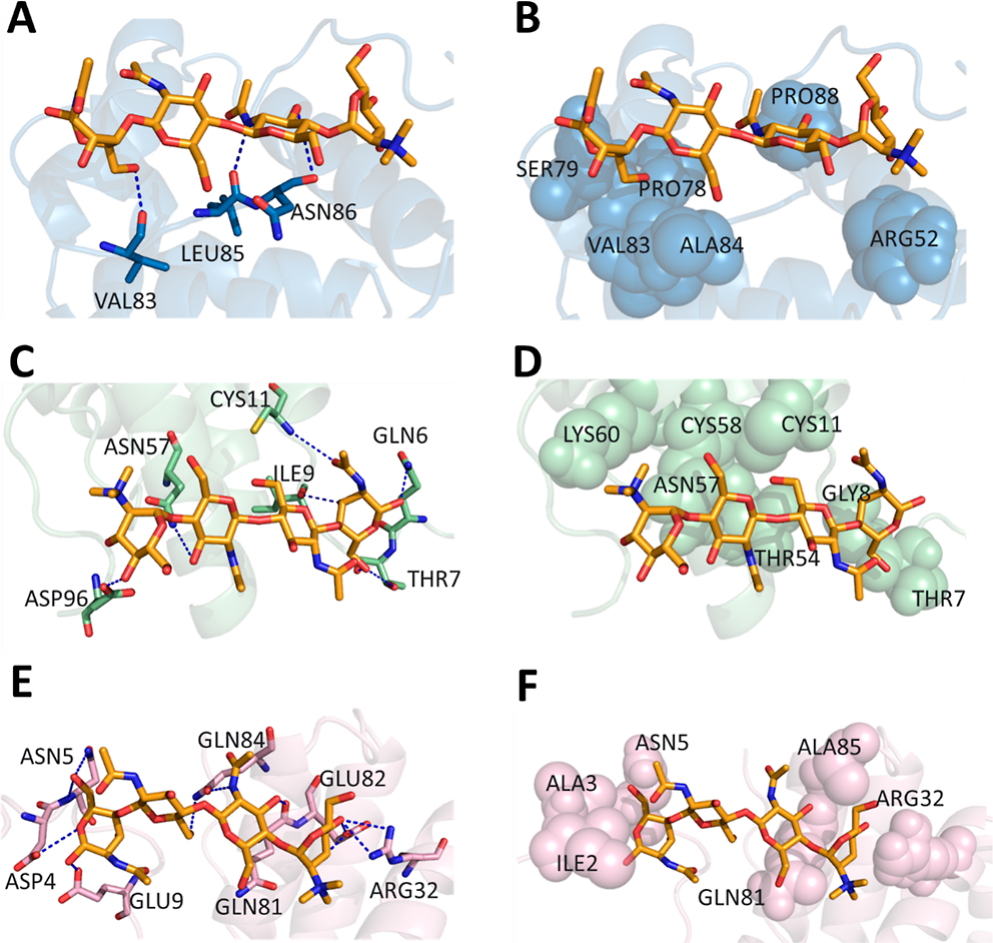
Docking of LTP with the tetramer of NAG_4_. Region of LTP: PHT58787—(NAG)_4_ interaction through (A) hydrogen bonds and (B) hydrophobic interactions. Region of LTP: A0A2G3AMC5—(NAG)_4_ interaction through (C) hydrogen bonds and (D) hydrophobic interactions. Region of LTP: A0A2G3AE21—(NAG)_4_ interaction through (E) hydrogen bonds and (F) hydrophobic interactions. Amino acid residues in lines participate in the interaction through hydrogen bonds; dashed blue lines are hydrogen bonds; amino acid residues in spheres participate in the interaction hydrophobically.

The three-dimensional LTP framework similar to *Cb*-F2 (accession: PHT58787) was put through a blind molecular docking experiment with (NAG)_4,_ and the best model showed negative values of affinity energy (−8.085 kcal/mol), indicating spontaneous binding (Figure 5A). Amino acid residues participating in the interaction with (NAG)_4_ VAL83, ALA84, LEU85, ASN86, and PRO88 are located in the 5° loop region. ARG52 is present in the 3^a^ α-helix, while PRO78 and SER79 are exposed in the 4^a^ α-helix of the model surface (Figure 5A). Docking experiment results showed that the LTP amino acids VAL83, LEU85, and ASN86 form hydrogen bonds with (NAG)_4_ (Figure 6A and Table 1). Hydrophobic interactions of LTP residues ARG52, PRO78, SER79, VAL83, ALA84, and PRO88 with (NAG)_4_ are represented in Figure 6B and Table 1.

**Table 1:** LTP Amino Acid Residues Involved in the Interaction with the NAG Tetramer

	PHT58787	A0A2G3AMC5	A0A2G3AE21
hydrogen bonds	VAL83	GLN6	ASP4
	LEU85	THR7	ASN5
	ASN86	ILE9	GLU9
		CYS11	ARG32
		ASN57	GLN81
		ASP96	GLU82
			GLN84
hydrophobic interactions	ARG52	THR7	ILE2
	PRO78	GLY8	ALA3
	SER79	CYS11	ASN5
	VAL83	THR54	ARG32
	ALA84	ASN57	GLN81
	PRO88	CYS58	ALA85
		LYS60	

The best docking of the LTP three-dimensional structure similar to *Cf*-F2 (accession: A0A2G3AMC5) with (NAG)_4_ also displayed a negative affinity energy value (−7.516 kcal/mol), indicating spontaneous binding (Figure 5B). Amino acid residues participating in the GLN6, THR7, GLY8, and ILE9 interactions are present in the first loop region of the protein model, while ASP96 is present in the last loop region. CYS11 is located in the 1^a^ α-helix, and the amino acid residues THR54, ASN57, CYS58, and LYS60 are in the 3^a^ α-helix exposed in the model surface (Figure 5B). The LTP–(NAG)_4_ complex suggests that the amino acid residues GLN6, THR7, ILE9, CYS11, ASN57, and ASP96 form hydrogen bonds with (NAG)_4_ (Figure 6C and Table 1). Amino acid residues THR7, GLY8, CYS11, THR54, ASN57, CSY58, and LYS60 interact hydrophobically with (NAG)_4_ (Figure 6D and Table 1).

Amino acid residues identified from *Cf*-F2 were also similar to LTP (accession: A0A2G3AE21). The docking performed showed a negative interaction energy (−7.749 kcal/mol), indicating spontaneous binding (Figure 5C). Amino acid residues participating in the interaction with (NAG)_4_ ILE2, ALA3, ASP4, ASN5, and GLU9 are present in the first loop region of the protein model. ARG32 is present in the 2^a^ α-helix, while GLN81, GLU82, GLN84, and ALA85 are present in the 6^a^ α-helix exposed in the model surface (Figure 5C). The results from docking experiments showed that amino acid residues ASP4, ASN5, GLU9, ARG32, GLN81, GLU82, and GLN84 form hydrogen bonds with (NAG)_4_ (Figure 6E and Table 1). Amino acid residues ILE2, ALA3, ASN5, ARG32, GLN81, and ALA85 interact with (NAG)_4_ through hydrophobic interactions (Figure 6E and Table 1).

### 3.5 Effect of *Cb*-F2 and *Cf*-F2 on the Growth of Yeast and Fungi

**Figure 7:**
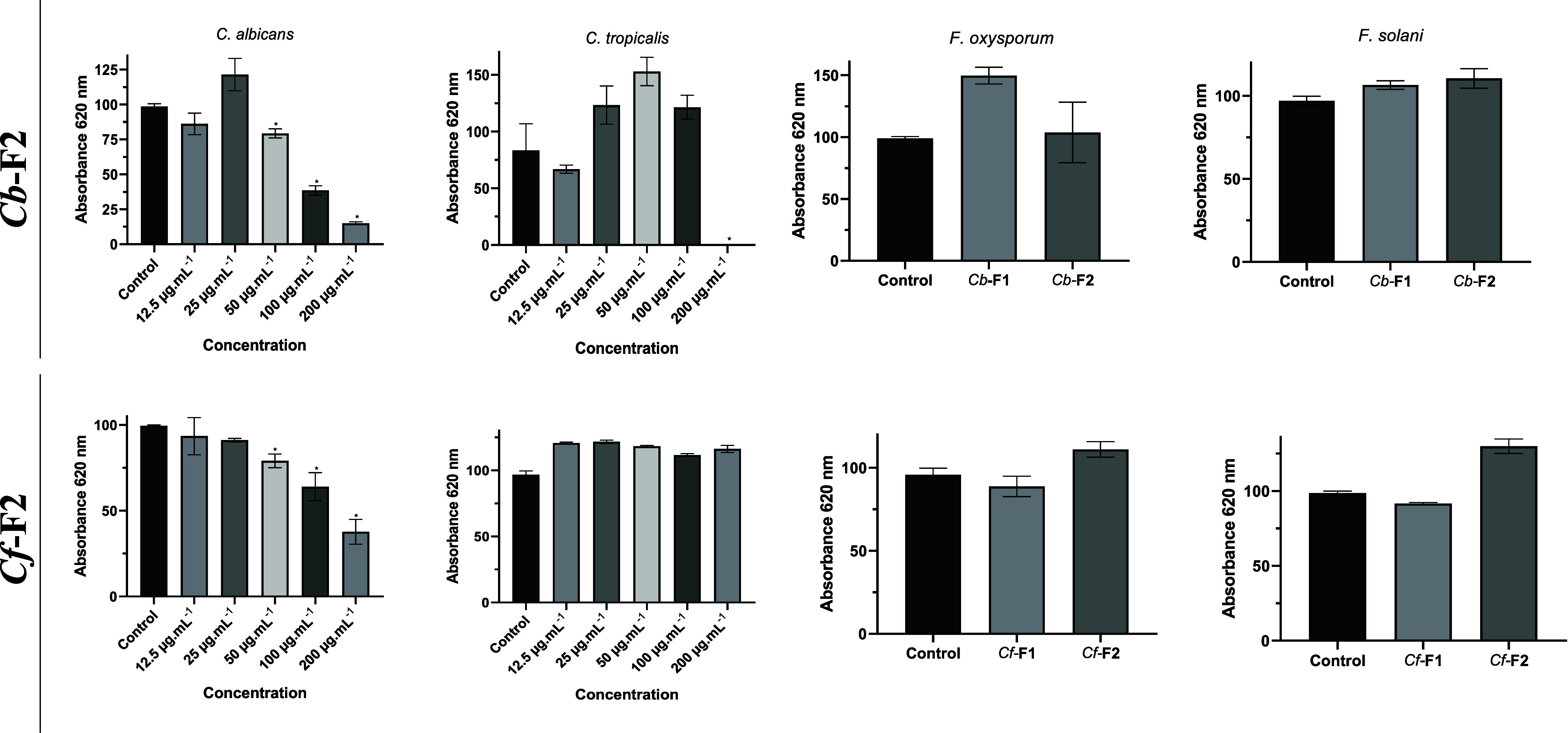
Effect of the *Cb*-F2 and *Cf*-F2 fractions from *C. baccatum* and *C. frutescens* on the growth of *C. albicans* and *C. tropicalis* at concentrations of 12.5, 25, 50, 100, and 200 μg.mL^–1^ for 24 h and the effect of *Cb*-F1, *Cf*-F1, *Cb*-F2, and *Cf*-F2 on the growth of *F. oxysporum* and *F. solani* at concentrations of 200 μg.mL^–1^ for 36 h. The values are the triplicates’ means (±SD). Significant changes (*p* < 0.05) between treatments and controls are indicated by asterisks. The growth inhibition % is shown by the values above the bars.

*Cb*-F2 inhibited the growth of *C. albicans* at concentrations of 50, 100, and 200 μg/mL^–1^ and inhibited the growth of *C. tropicalis* only at a concentration of 200 μg/mL^–1^; however, it inhibited 100% of the growth of yeast. *Cf*-F2, on the other hand, only inhibited the growth of the yeast *C. albicans* at concentrations of 50, 100, and 200 μg/mL^–1^. It was also found that the retained (*Cb*-F2 and *Cf*-F2) and nonretained (*Cb*-F1 and *Cf*-F1) fractions did not show activity against phytopathogenic fungi at the tested concentrations (Figure 7).

### 3.6 Cell Viability

**Figure 8:**
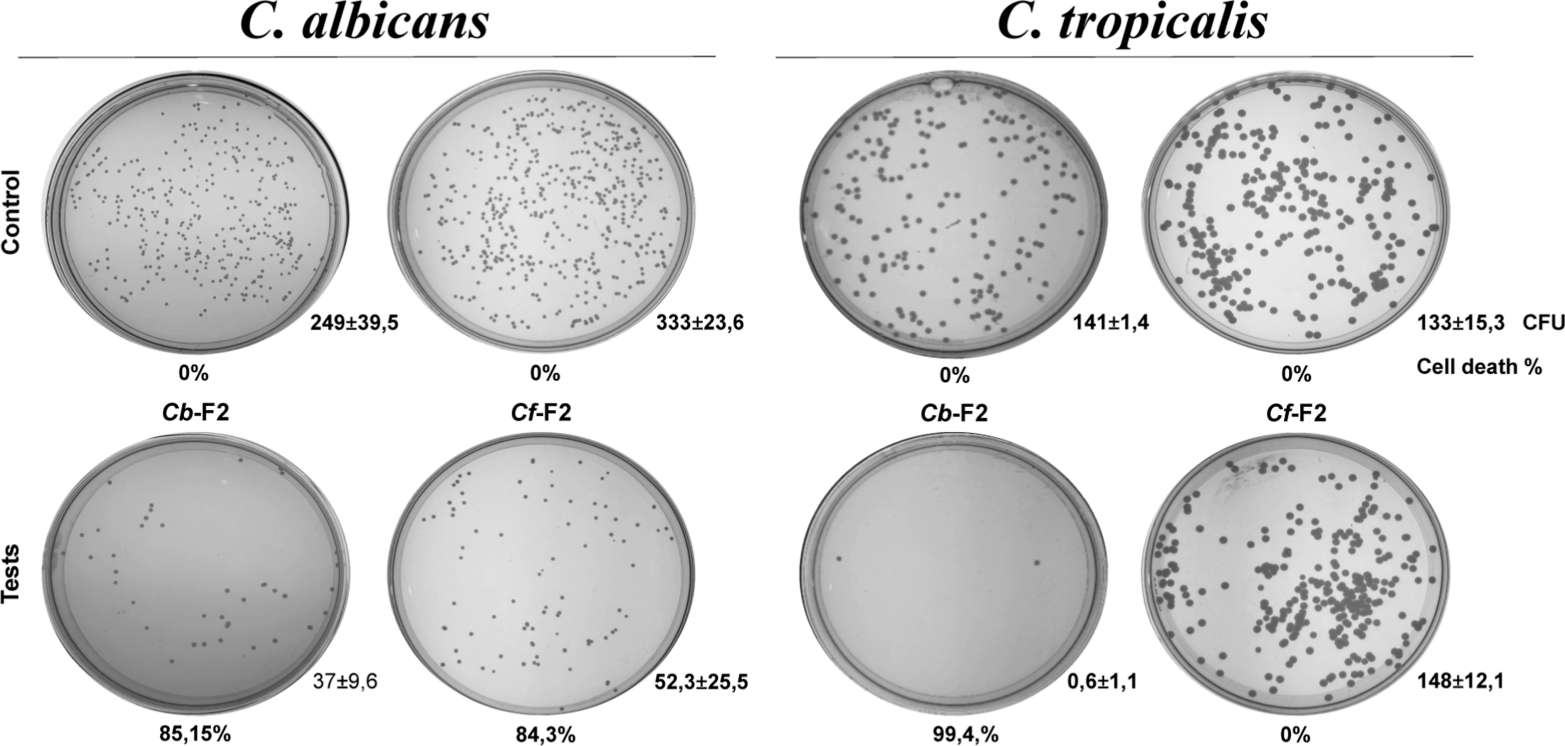
Cell viability of the yeasts *C. albicans* and *C. tropicalis*’. Images of the colony growth in control settings captured in Petri dishes (without the addition of the F2 fraction) and after treatment with 200 μg.mL^–1^ of *Cb*-F2 and *Cf*-F2 fractions for 36 h. Relative to the untreated control cells, the percentage of cell death was computed (cell viability—100%). Three duplicates of each experiment were carried out. CFU, colony-forming unit; numbers beneath the images show the cell death percentage.

Due to the antimicrobial activity of the fractions, the cell viability of the yeasts *C. albicans* and *C. tropicalis* was determined at 200 μg/mL^–1^ with an incubation period of 36 h (Figure 8). *Cb*-F2 decreased the quantity of units that form colonies (CFU) for *C. albicans*, showing 85.15% loss of viability. For *C. tropicalis*, the fraction showed an even greater reduction of 99.4%, indicating a fungicidal effect for this species. *Cf*-F2, on the other hand, showed a lethal dose of 84.3% for *C. albicans*. As expected, there was no CFU reduction for *C. tropicalis*.

### 3.7 Toxicity Effect on *G. mellonella* Larvae of *Cb*-F2 and *Cf*-F2

**Figure 9:**
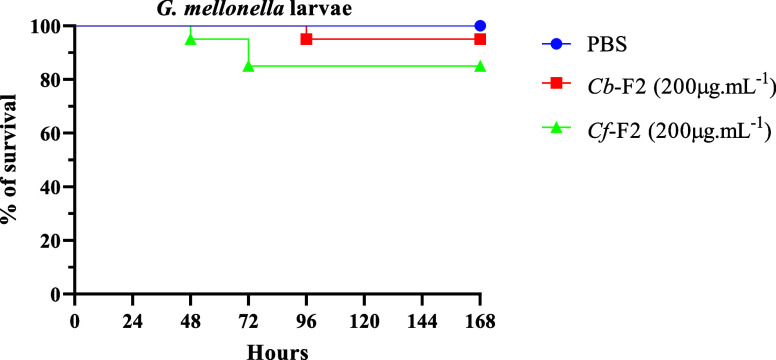
Effect of *Cb*-F2 and *Cf*-F2 in vivo on *G. mellonella* larvae. Survival curves were generated for *G. mellonella* larvae treated with *Cb*-F2 and *Cf*-F2 at 200 μg.mL^–1^. Controls included PBS and needle wounds. Statistical significance was determined using the Gehan–Breslow–Wilcoxon test with a significance level set at *p* < 0.05.

The ability of *Cb*-F2 and *Cf*-F2 to cause toxicity in *G. mellonella* larvae at a concentration of 200 μg/mL^–1^ was evaluated. The larvae that were inoculated with both fractions showed a low level of toxicity. For *Cb*-F2, it was verified that at the tested concentration, there was a 95% survival rate in larvae. For the *Cf*-F2 fraction, it was verified that at the same concentration, there was a lower survival rate, at 85% (Figure 9).

## 4 Discussion

This work describes the extraction of proteins from seeds of two species of the genus *Capsicum*: *C. baccatum* (accession UENF 1732) and *C. frutescens* (accession UENF 1775). After extraction, the peptide-rich fractions obtained by heating the extract were purified by affinity chromatography on a chitin column, resulting in two fractions for both species, namely, *Cb*-F1 (fraction not retained) and *Cb*-F2 (fraction retained in the chitin column) for *C. baccatum* and *Cf*-F1 (fraction not retained) and *Cf*-F2 (fraction retained) for *C. frutescens* (Figure 1A,C). To verify the protein profiles, tricine–SDS–PAGE was performed (Figure 1B,D) for both fractions of both species. All fractions showed low molecular mass bands, especially those that bound to the chitin column (F2). *Cb*-F2 presented two major bands between 3.4 and 14.2 kDa, while *Cf*-F2 presented three major bands between 6.5 and 14.2 kDa. Other studies reported the presence of chitin-binding peptides isolated from plant seeds, such as Fa_AMP1 and Fa_AMP2, extracted from *Fagopyrum esculentum,*(47) and Mo-CBP4 extracted from *Moringa oleifera*.^6^ In the case of species of the genus *Capsicum*, little is known about chitin-binding peptides and their possible activities.

Mass spectrometry sequencing of the bands of the *Cb*-F2 and *Cf*-F2 fractions showed similarity with the proteins of the LTP family (Figure 3). The presence of peptides related to the LTP family was also confirmed by Western blotting (Figure 2). This result shows for the first time the property of LTPs to associate with the chitin matrix. The relevance of this finding will be investigated later.

Other works reported the isolation and characterization of nsLTPs, which involve extraction of proteins with saline buffer or acidic solution, fractionation in ammonium sulfate (∼80%),^26,48−50^ and subsequent chromatography, which may include ion exchange (DEAE-Sephadex), size exclusion chromatography (Sephadex G-50), or reversed-phase high-performance liquid chromatography (RP-HPLC).^13,48,51,52^ This is the first work that reports the isolation of LTPs through chitin affinity chromatography.

The most conserved region is the 8CM motif, and the distinction between the types of LTP is delineated by the nature of the disulfide bonds, with variation in the C5 and C6 cysteines, leading to functional effects on the tertiary structure. One distinguishing feature of type I nsLTPs compared to others is the presence of a conserved glycine between helices 1 and 2, linked by a disulfide bridge between C2 and C3. It is also known that lysine and tyrosine residues exhibit conservation among type I nsLTPs. These disparities contribute to the structural variances and may influence their respective mechanisms.^53^

Generally, the three-dimensional structure of nsLTPs presents a condensed domain comprising four helices (H1–H4) linked by brief loops (L1–L3) and an unstructured C-terminal tail.^52^ Helices 1 and 2 and the C-terminus are generally longer in type I nsLTPs. The domain is joined by several intramolecular hydrogen bonds and by four disulfide bridges conserved in the patterns: (1): C1–C6, C2–C3, C4–C7, and C5–C8 for type I nsLTPs^54^ and (2): C1–C5, C2–C3, C4–C7, and C6–C8 for type II nsLTPs.^55^ Furthermore, the three-dimensional structures have an internal tunnel-shaped cavity that will accommodate different types of lipids, in addition to being stable against thermal and digestive processing.^56^

The LTP three-dimensional structure similar to *Cb*-F2 (accession: PHT58787), *Cf*-F2 (accession: A0A2G3AMC5), and *Cf*-F2 (accession: A0A2G3AE21) isolated from *C. baccatum* and *C. frutescens*, respectively, was put through a blind molecular docking experiment using (NAG)_4_. The docking of LTP with (NAG)_4_ showed negative affinity energy values (−8085 kcal/mol), (−7516 kcal/mol), and (−7749 kcal/mol), respectively, for the previously described accessions, demonstrating the hydrophobic and hydrogen-bond interactions that occur spontaneously. The amino acids participating in the interaction with (NAG)_4_ were VAL83, ALA84, LEU85, ASN86, PRO88, ARG52, PRO78, and SER79 for the first access, GLN6, THR7, GLY8, ILE9 ASP96, CYS11, THR54, ASN57, CYS58, and LYS60 for the second access, and ILE2, ALA3, ASP4, ASN5, GLU9, ARG32, GLN81, GLU82, GLN84, and ALA85 for the third access.

In a previous work, Ventury et al.^57^ additionally showed molecular docking with the vicilin protein, nevertheless. It was demonstrated that vicilin presented spontaneous binding, with energy values of (−7527 kcal/mol), involving interactions through hydrogen bonds, salt bridges, and hydrophobic forces. In another study carried out by Nazeer et al.,^26^ LTP was isolated from *T. ammi* seeds. The projected three-dimensional structure of the isolated protein includes a lengthy C-terminal tail and four α-helices supported by four disulfide links. Docking was also carried out with two different ligands (myristic acid and oleic acid). The amino acids Leu11, Leu12, Ala55, Ala56, Val15, Tyr59, and Leu62 are suggested to be essential for the binding of lipid molecules. Numerous 3D structures of LTPs that have been solved have demonstrated the variability of this binding pattern involving certain residues with ligands. In this study, we evaluated whether *Cb*-F2 and *Cf*-F2 were capable of inhibiting the growth of the yeasts *C. albicans* and *C. tropicalis* and the phytopathogens *F. oxysporum* and *F. solani*. We observed that *Cb*-F2 inhibited the growth of both yeasts; however, *Cf*-F2 only inhibited the growth of the yeast *C. albicans*. Regarding phytopathogens, both fractions were unable to inhibit the growth of the two fungi (Figure 7). To be considered fungicidal, an antifungal must reduce the number of colonies by 99% in CFU.mL, and to be considered fungistatic, it must reduce the number of colonies by <99% in CFU.mL in relation to the initial inoculum.^58^

Currently, there are four main classes of commercially used antifungals, polyenes, pyrimidine analogues, azoles, allylamines, and echinocandins. Polyenes are the oldest class among these antifungals, with amphotericin B (AmB) being the best-known member of this class.^59^ Souza et al.^60^ carried out an AmB growth inhibition assay on *C. albicans* yeast. It can be observed that the antifungal inhibited 100% of yeast growth at concentrations of 1.56, 0.78, 0.39, 0.19, and 0.09 μM. However, despite having one of the most potent antifungal compounds, their use is limited due to their severe nephrotoxicity and low solubility in water. The most commonly used antifungals are azoles. They are divided into two classes, the imidazoles (ketoconazole and miconazole) and the triazoles (fluconazole (FLC), itracolazole, posaconazole, voriconazole, and isavuconazole).^59^ Taveira et al.^61^ carried out a growth inhibition assay with FLC on the yeasts *C. albicans*, *C. tropicalis*, *Candida parapsilosis*, and *Candida pelliculosa*. It was found that the necessary concentration of the antifungal to inhibit 50% of yeast growth was, respectively, 1.0, 1.0, 0.5, and 5.0 μg.mL^–1^. However, it is known that azoles suffer from the rapid development of resistance and tolerance to several species. Furthermore, they are subject to unfavorable drug interactions.^59^

nsLTPs have been the subject of several antimicrobial studies. Although their in vivo studies are little explored, it is known that they are capable of inhibiting the growth of important pathogenic microorganisms in vitro. For example, nsLTPs from *C. anuumm* seeds demonstrated activity against *Colletotrichum lindemunthianum* and *C. tropicalis,* with efficacy demonstrated at a concentration of 400 μg/mL^–1^ for both species. This concentration corresponds to a lethal dose for 70% of *C. tropicalis* cells. In contrast, when using *Cb*-F2, we observed 100% cell mortality using only half the concentration (200 μg/mL^–1^) compared to *C. annuum* nsLTP.^62^ In another study with the same species, the authors demonstrated that the identified LTP had activity against *Saccharomyces cerevisiae*, *Pichia membranifaciens*, *C. tropicalis*, and *C. albicans*,^63^ while the LTP identified in *Coffea canephora* demonstrated activity against *C. albicans* at a concentration of 400 μg/mL^–1^, inhibiting only 50% of cell growth,^64^ while *Cb*-F2 and *Cf*-F2 inhibited 84 and 62% at a concentration of 200 μg/mL^–1^, respectively.

The release of nsLTP into the apoplast is probably a defense reaction mechanism, permitting its connection with molecules secreted by microorganisms.^17^ Consequently, they interact with receptors such as serine/threonine protein kinases (PKs), which possess a transmembrane region, a cytoplasmic PK, and an extracellular leucine-rich repeat domain. This interaction initiates a cascade of protein kinases (MAPKs), inducing protective factors, such as PR proteins (related to pathogenesis), AMPs, and SARs (acquired systemic resistance).^53^

The toxicity of fractions on *G. mellonella* larvae was assessed in this study. These larvae are used as an alternative to traditional experimental models that use murines, as there are positive correlations between the results obtained in *G. mellonella* and murine models. This approach reduces experimental time and costs in addition to improved ethics considerations in animal welfare.^65^ We can observe that both fractions demonstrated low toxicity at a concentration of 200 μg/mL^–1^, especially the *Cb*-F2 fraction, presenting survival rates of 95 and 85% for *Cb*-F2 and *Cf*-F2, respectively (Figure 9). Although the peritrophic membrane (PM) present in the midgut of larvae is rich in chitin, we found that at this concentration, the fractions do not have the capacity to fully bind to this matrix and rupture it. Other studies report that chitin-affinity proteins can interfere with insect PM. The CBP fraction was able to interfere with the development of *Callosobruchus maculatus*, reducing larval mass and length,^66^ whereas CBPA present in *Bacillus thuringiensis* helped direct the bacteria to the PM of *G. mellonella* larvae.^67^ Since chitin-binding proteins have been shown to interfere with PM, more testing at various dosages is necessary to verify their minimal toxicity.

Infectious diseases and problems with antimicrobial resistance are increasingly raising a demand for novel antimicrobial compounds that have a wide range of action and minimal negative effects.^68^ As a result, AMPs have been investigated as a possible substitute for conventional antibiotics, including nsLTPs, which have several actions, such as antibacterial, antiviral, enzymatic, and antifungal activities.^69^ Its positive charge encourages it to attach to molecules that are negatively charged, such as lipopolysaccharides and phospholipids, which cause cell death.^16^ As seen in this work, the *Cb*-F2 and *Cf*-F2 fractions are promising candidates for studying the development of effective methods for fungal control. However, further studies must be carried out to improve the results obtained.

## 5 Conclusions

Two fractions of the species *C. baccatum* and *C. frutescens* were identified after chitin affinity chromatography, and their in vitro and in vivo activities were evaluated. Both fractions demonstrated in vitro antimicrobial activity against *C. albicans*, and only the *Cf*-F2 fraction did not show activity against *C. tropicalis*. After mass spectrometry, it was verified that both fractions were similar to LTPs, a group that has several functions in plants, including antifungal action. The toxicity of the fractions in vivo in *G. mellonella* larvae was also evaluated. It was verified that at the concentration tested, neither fraction presented a toxic effect on the larvae, indicating that they are candidates for the development of new therapeutic agents.

## Data Availability

All data generated or analyzed during this study are included in this published article.
